# Hydroperitoneum: A Rare Complication Performing Endoscopic Combined Intrarenal Surgery

**DOI:** 10.1089/cren.2016.0024

**Published:** 2016-03-01

**Authors:** Alfonso Benincasa, Federico Nicolosi, Mario Falsaperla, Alberto Saita

**Affiliations:** Department of Urology, Vittorio Emanuele Hospital, Catania, Italy.

## Abstract

***Background:*** Recently endoscopic combined intrarenal surgery (ECIRS) has been introduced as an innovative approach for the treatment of complex single, multiple, and staghorn urolithiasis, which reveals to be a viable alternative to standard percutaneous nephrolithotomy. Although considered to be a versatile, safe, and efficient endoscopic procedure, it is not free from complications. We would like to report two rare cases of hydroperitoneum that occurred during ECIRS and their management.

***Case Presentation:*** Two female patients, respectively, of 75 and 41 years of age, underwent ECIRS procedure for the treatment of complex staghorn kidney urolithiasis, previously evaluated by noncontrast computed tomography (CT) scan. A combined retrograde-percutaneous access to the intrarenal collecting system, under fluoroscopic and ultrasound guidance with the additional assistance of Endovision technique, was performed. At the end of the procedures, both patients revealed a taut and globous abdomen, and a perioperative CT and ultrasonography revealed the presence of intraperitoneal liquid. Both patients were effectively treated with immediate positioning of abdominal drain with rapid improvement of the clinical presentation.

***Conclusion:*** To our knowledge these are the first two cases of hydroperitoneum occurring during ECIRS and reported in the literature. An early detection of the complication and its prompt treatment revealed to be crucial to effectively prevent morbidity.

## Clinical History

The first patient, a 75-year-old female, presented to our institution with left staghorn renal stone associated with left lumbar pain and flank irradiation and no other related complications. Her medical history included Alzheimer and pulmonary emphysema. Her prior surgeries included appendicectomy, cholecystectomy, and thyroidectomy for non-neoplastic disease; no previous history of stone disease had been documented. The second patient, a 41-year-old female, showed right staghorn urolithiasis involving renal pelvis, upper and inferior caliceal system associated with moderate hydronephrosis, flank pain, and *Enterobacter* recurrent urinary tract infections. Her prior surgery history revealed right retrograde intrarenal surgery (RIRS) performed in another institution, which did not totally solve the problem of the lithiasis. No other relevant pathologic findings emerged from her medical history. In both patients, preoperative renal function did not appear compromised.

## Physical Examination

They, respectively, were 160 and 168 cm tall and weighed 50 and 78 kg; their BMI was 19.5 and 27.8 kg/m^2^, respectively. The abdomen was soft in both cases, globous for adiposity in the second case. Blood pressures were normal as the other remaining vital signs. No remarkable skeletal abnormalities emerged.

## Diagnostic Studies

In both patients preoperative noncontrast computed tomography (CT) scan was performed. In both cases, the examination revealed a complex staghorn stone involving renal pelvis and the upper and middle caliceal system, associated with mild dilation of the urinary tract. Stones measured, respectively, about 35 and 28 mm, Hounsfield units were 955 and 1108, respectively. Infundibulopelvic angle (measured as inner angle formed at intersection of ureteropelvic axis and central axis of lower pole infundibulum) gauged 63 and 65 degrees and stone burden (length × width × 0.25 × 3.14) was about 380 and 320 mm^2^, respectively. Urine analysis and urine culture were negative in the first patient, whereas in the second patient they showed an infection substained by *Escherichia coli*, and a prompt antibiotic therapy, based on specific antibiogram, was carried out. Other laboratory parameters did not show any pathologic findings.

## Intervention

Endoscopic combined intrarenal surgery (ECIRS) procedures were planned and performed by the same first surgeon who was already experienced in prone percutaneous nephrolithotomy (PCNL) and RIRS amounting to 280 cases in total, 90 of which were ECIRS. The first patient was placed in a prone position, whereas the second patient was placed in Galdakao-modified supine Valdivia position, combining the supine position of the patient with the flank elevated and a modified lithotomic arrangement of the lower limbs, the ipsilateral one extended and the contralateral one well abducted.^1^ Irrigant fluid (0.9% sodium chloride) was located at 50 cm above patient level to avoid high intraluminal pressures. Cystoscopy was performed using 22F cystoscope (Karl Storz-Endoskope^®^); retrograde pyelography, conducted with ureteral catheter 6F (RUSH^®^) and integrated by fluoroscopic guidance that excluded the presence of ureteral strictures, malformations, or stone fragments, confirmed in both cases the stone characteristics previously evaluated by CT scan.

A 0.035′′ hydrophilic guide (Boston Scientific Sensor^®^) was placed into the ureteral lumen. Subsequently, semirigid ureteroscopy was carried out using 8F ureteroscope (Karl Storz-Endoskope) using a second guidewire. A 10 to 12 mm ureteral sheath (Coloplast Re-Trace^®^) was then located and flexible renoscopy with 8F flexible ureteroscope FLEX-X^2^ (Karl Storz-Endoskope) was conducted. Percutaneous renal access was carried out by puncturing the lower posterior kidney calix with Chiba needle 18-gauge under biplanar fluoroscopic and ultrasound guidance with also the additional assistance of the Endovision technique. Intraoperative urine samples for cultures from the upper urinary tract were systematically obtained. Upon insertion of guidewire 0.035′′ (BARD Black wire/ultratorque^®^) through the 18-gauge needle sent down the ureter and the bladder and exiting through the external meatus, percutaneous tract was dilated to 24F using balloon (BARD X-Force^®^) and then Amplatz working sheath 24F was located.

**FIG. 1.**
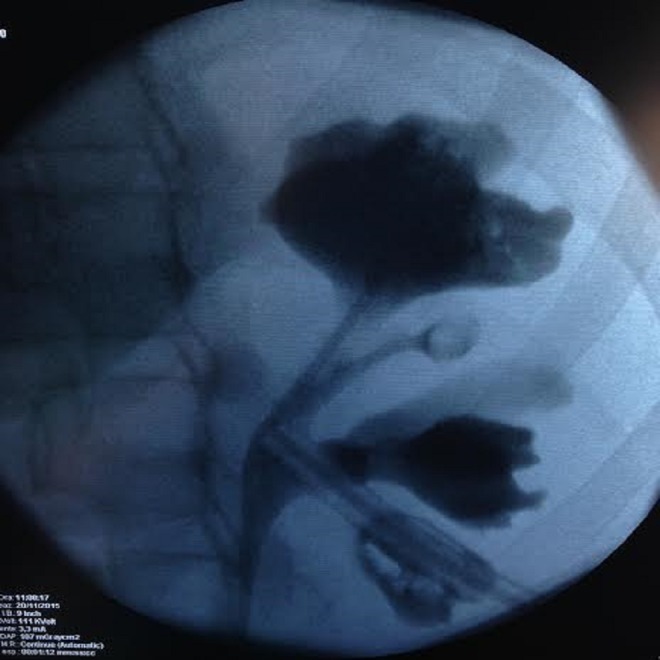
Postoperative pyelography.

Stones were disintegrated using ballistic energy, with combined ballistic and ultrasonic (SWISS LITHOCLAST^®^ MASTER) or ballistic and Holmium laser energies (DORNIER Medilas H20^®^). We used Nephroscope 22F (Karl Storz-Endoskope) to ensure a good outflow of the irrigation liquid between the Amplaz sheath and the nephroscope during the procedure. Stone fragments were extracted using nitinol basket 1.9F (Zero Tip^®^ Boston Scientific) and extractors 10F (Perc N-Circle^®^ COOK). At the end of both procedures, a Double-J ureteral stent 6F (Polaris Ultra Boston Scientific^®^) and nephrostomy tube 8F (Soft Drain Bard^®^) were inserted. Postoperative pyelography did not show any contrast leakage outside the collecting system and outside the ureter after the removal of the ureteral access sheath in both cases (Fig. 1).

**FIG. 2.**
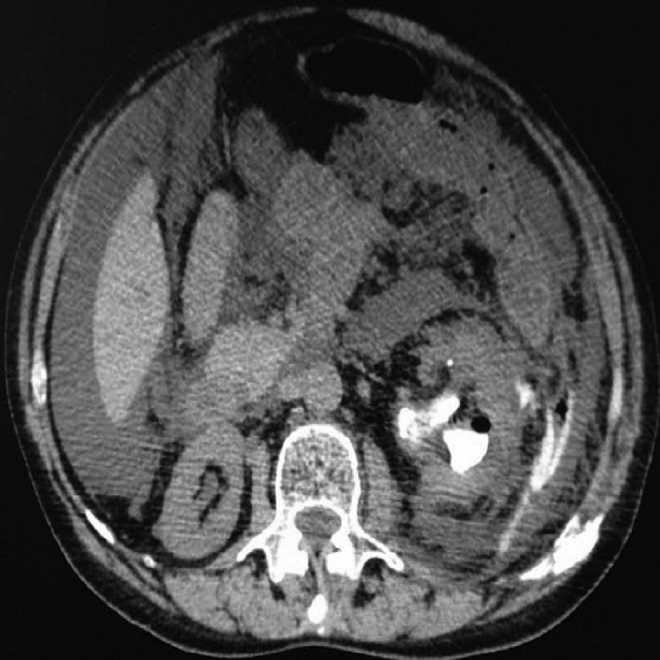
Postoperative CT scan showing intraperitoneal fluid collection.

**FIG. 3.**
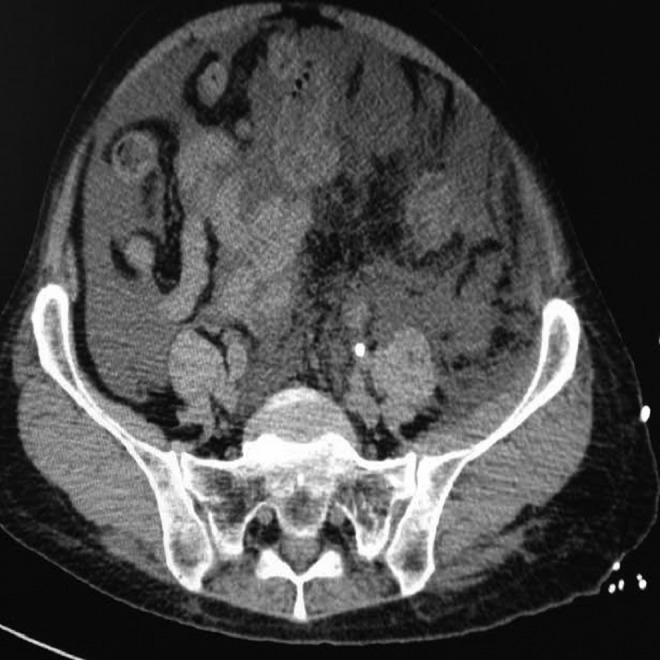
Postoperative CT scan showing intraperitoneal fluid collection.

**FIG. 4.**
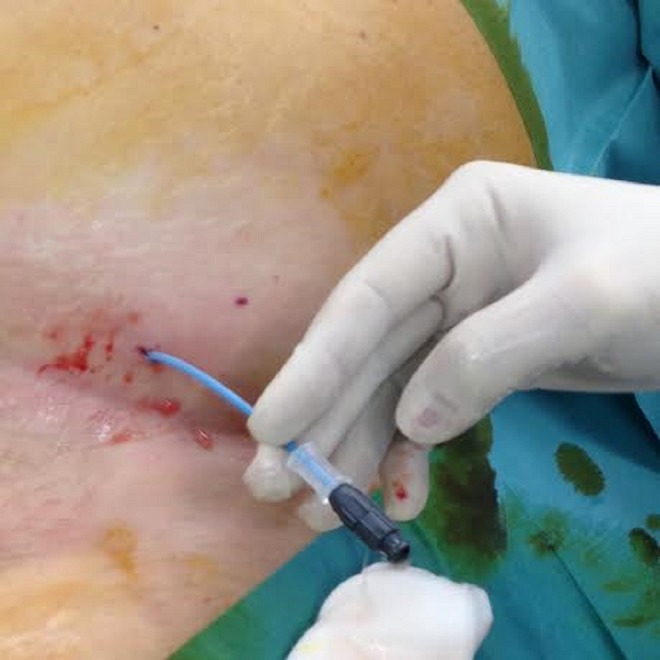
Abdominal drain.

Operative time was about 90 minutes in the first case and about 110 minutes in the second case. At the end of both procedures, patients revealed a taut and globous abdomen. In the first case, a CT scan under anesthesia was performed; in the second case, an abdominal ultrasonography was carried out because of the unavailability of CT malfunction in our institution, both showing an abundant collection of intraperitoneal fluid (Figs. 2 and 3). Both patients were effectively treated with an immediate positioning of abdominal drain pigtail 8F (Soft Drain BARD) (Fig. 4), respiratory and blood gas monitoring. About 1.5 L of fluid was drained from each patient with a rapid improvement of their clinical presentation.

## Outcome

Postoperative laboratory studies showed stable levels of hemoglobin, hematocrit, platelets, and electrolytes; renal function, evaluated with bcreatinine and blood urea nitrate, revealed preserved. Only in the first patient, postoperative hypokalemia occurred and was treated with intravenous administration of potassium chloride.

CT scan and ultrasonography 2 days after ECIRS showed complete resolution of staghorn stone and of the hydroperitoneum. Abdominal drains were removed 24 hours after surgery and nephrostomy tubes were removed 5 days after surgery in both patients, always preceded by a pyelography checkup.

## Discussion and Literature Review

ECIRS, in prone and supine position, is widely accepted to be a viable alternative to the only PCNL for the treatment of complex urolithiasis.^2,3^ Stone-free rate achieved is about 81.9% after first treatment and is about 87.4% after a second procedure as reported in the literature, equaling data reported by PCNL, ranging from 67% to 96.5%.^4^ It also offers more anesthesiologic and urologic advantages, including a greater stone manipulation allowing the combined percutaneous and retrogade access, a better drainage, and fragments removal; it also avoids multiple accesses and related morbidity and consents and easier patient monitoring. Although considered a versatile, safe, and efficient endoscopic procedure, it is not free from complications. According to the literature, the total complication rate, for both techniques, was found around 83% mostly classified as Clavien grade 1–2.^5^

Among many complications observed, no previous reports of hydroperitoneum emerged from literature review and we wanted to provide the best management of a complication that apparently may seem complex to deal with. Positioning of an abdominal drain under CT or ultrasound guidance revealed to be easy and safe, avoiding other organ injuries and ensuring a rapid resolution of symptoms. We do not know exactly the reason for this complication. The same surgical technique was used for all previous ECIRS procedures. Lower posterior kidney calix was reached on the first attempt to puncture, and the contrastographic controls, carried out during and after the dilatation of the percutaneous tract, did not highlight any evident injury of the pyelo-caliceal system. We can only suppose that there has been an osmotic liquid filtration from the extraperitoneal to the intraperitoneal compartment since postoperative pyelography did not show any extra renal leakage of contrast medium and/or direct linking with the peritoneal cavity.

## Conclusion

To our knowledge, these are the first two cases of hydroperitoneum that occurred during ECIRS and reported in the literature. An early detection of the complication and its prompt treatment revealed to be crucial to effectively prevent morbidity.

## Abbreviations Used

CT: computed tomography
ECIRS: endoscopic combined intrarenal surgery
PCNL: percutaneous nephrolithotomy
RIRS: retrograde intrarenal surgery
